# Fate of Astrocytes in The Gerbil Hippocampus After Transient Global Cerebral Ischemia

**DOI:** 10.3390/ijms20040845

**Published:** 2019-02-15

**Authors:** Hyeyoung Kim, Joon Ha Park, Myoung Cheol Shin, Jun Hwi Cho, Tae-Kyeong Lee, Hyunjung Kim, Minah Song, Cheol Woo Park, Young Eun Park, Jae-Chul Lee, Sungwoo Ryoo, Young-Myeong Kim, Dae Won Kim, In Koo Hwang, Soo Young Choi, Moo-Ho Won, Ji Hyeon Ahn

**Affiliations:** 1 Department of Emergency Medicine, School of Medicine, Kangwon National University, Chuncheon, Gangwon 24341, Korea; hae1127@hanmail.net (H.K.); dr10126@naver.com (M.C.S.); cjhemd@kangwon.ac.kr (J.H.C.); 2 Department of Anesthesiology and Pain Medicine, Chungju Hospital, Konkuk University School of Medicine, Chungju, Chungcheongbuk 27376, Korea; 3 Department of Biomedical Science and Research Institute for Bioscience and Biotechnology, Hallym University, Chuncheon, Gangwon 24252, Korea; jh-park@hallym.ac.kr (J.H.P.); sychoi@hallym.ac.kr (S.Y.C.); 4 Department of Neurobiology, School of Medicine, Kangwon National University, Chuncheon, Gangwon 24341, Korea; xorud312@naver.com (T.-K.L.); nicolehkim@naver.com (H.K.); zlscydn@naver.com (M.S.); flfhflfh@naver.com (C.W.P.); taeparo@naver.com (Y.E.P.); anajclee@kangwon.ac.kr (J.-C.L.); 5 Department of Biological Sciences, College of Natural Sciences, Kangwon National University, Chuncheon, Gangwon 24341, Korea; ryoosw08@kangwon.ac.kr; 6 Department of Molecular and Cellular Biochemistry, School of Medicine, Kangwon National University, Chuncheon, Gangwon 24341, Korea; ymkim@kangwon.ac.kr; 7 Department of Biochemistry and Molecular Biology, and Research Institute of Oral Sciences, College of Dentistry, Gangnung-Wonju National University, Gangneung, Gangwon 25457, Korea; kimdw@gwnu.ac.kr; 8 Department of Anatomy and Cell Biology, College of Veterinary Medicine, and Research Institute for Veterinary Science, Seoul National University, Seoul 08826, Korea; vetmed2@snu.ac.kr

**Keywords:** astrogliosis, delayed neuronal death, Fluoro Jade B, transient global ischemia, hippocampus

## Abstract

Neuronal death and reactive gliosis are major features of brain tissue damage following transient global cerebral ischemia (tgCI). This study investigated long-term changes in neuronal death and astrogliosis in the gerbil hippocampus for 180 days after 5 min of tgCI. A massive loss of pyramidal neurons was found in the hippocampal CA1 area (CA1) area between 5 and 30 days after tgCI by Fluoro-Jade B (FJB, a marker for neuronal degeneration) histofluorescence staining, but pyramidal neurons in the CA2/3 area did not die. The reaction of astrocytes (astrogliosis) was examined by glial fibrillary acidic protein (GFAP) immunohistochemistry. Morphological change or degeneration (death) of the astrocytes was found in the CA1 area after tgCI, but, in the CA2/3 area, astrogliosis was hardly shown. GFAP immunoreactive astrocytes in the CA1 area was significantly increased in number with time and peaked at 30 days after tgCI, and they began to be degenerated or dead from 40 days after tgCI. The effect was examined by double immunofluorescence staining for FJB and GFAP. The number of FJB/GFAP^+^ cells (degenerating astrocytes) was gradually increased with time after tgCI. At 180 days after tgCI, FJB/GFAP^+^ cells were significantly decreased, but FJB^+^ cells (dead astrocytes) were significantly increased. In brief, 5 min of tgCI induced a progressive degeneration of CA1 pyramidal neurons from 5 until 30 days with an increase of reactive astrocytes, and, thereafter, astrocytes were degenerated with time and dead at later times. This phenomenon might be shown due to the death of neurons.

## 1. Introduction

It has well been investigated that 5 min of transient global cerebral ischemia (tgCI) selectively causes delayed neuronal death (DND) of pyramidal neurons in the CA1 area of the hippocampus from 4 to 5 days after tgCI, whereas pyramidal neurons in the CA2/3 area remain intact in gerbils [1,2]. It is well known that DND is accompanied by gliosis, which is characterized by reactions of astrocytes and microglia [3,4]. Astrocytes are the most abundant glial cells in the central nervous system (CNS) and support functions for neurons, such as K^+^ buffering, H^+^ control, neurotransmitters uptake, blood–brain barrier regulation, and water transport, in the CNS [5]. In ischemic brains, astrocytes respond to ischemic injury to restore homeostasis. On the other hand, they are involved in the production of pathogenic substances [6] and selective dysfunction of astrocytes, which is implicated in neuronal loss after ischemic insults [7].

Astrocyte activation or reactive astrogliosis is defined as a constitutive, graded, multi-stage, and evolutionary conserved defense. This is accompanied by upregulation of glial fibrillary acidic protein (GFAP) and hypertrophy of astrocytes’ processes, and the role of activated astrocytes has been understood to be involved in pathogenesis and the recovery process in damaged CNS areas [6,8]. The reactivity of GFAP depends on the degree of damage and the distance between astrocytes and the injured area [9]. For example, serious ischemic damage following a longer ischemic duration or under higher temperature induces much severer astrocyte activation in damaged sites [10].

The dysfunction or death of astrocytes after stroke has been studied using astrocytic proteins, such as GFAP, vimentin, S100, glutathione-S-transferase Y_b_ (GST Y_b_), combined with markers of cell damage/death, such as in situ terminal deoxynucleotidyl transferase dUTP nick end labeling (TUNEL) [11], in situ DNA polymerase I dATP nick translation (PANT) [12] or in situ end labeling (ISEL) [13], to detect DNA fragmentation, and caspase-3 and caspase-12 [11] to detect caspase activation (a form of programmed cell death). In addition, Fluoro-Jade B (FJB) histofluorescence staining specifically detects degenerating neurons in the CNS following cerebral ischemia [14,15]. Recent studies have shown that FJB detects quiescent astrocytes in the cerebral cortex of a primate model of Alzheimer’s disease [16] and in the spinal cord of a rat model of traumatic injury [17].

Many studies have reported necrotic tissues (infarctions) responded to focal ischemia in the early period after focal ischemia and astrocyte impairment in animal models of transient ischemic insults; however, studies on the chronic change of astrocytes in response to transient global cerebral ischemia (tgCI) have not been reported yet. Therefore, the purpose of this study was to investigate the long-term pattern of astrocyte reaction in the hippocampal CA1 area (CA1), which is most vulnerable to tgCI, after tgCI in gerbils. In addition, we compared it with that in the CA2/3, which is resistant to tgCI in gerbils, which are an excellent animal model of tgCI [18,19]. 

## 2. Results

### 2.1. CV Staining

**Figure 1:**
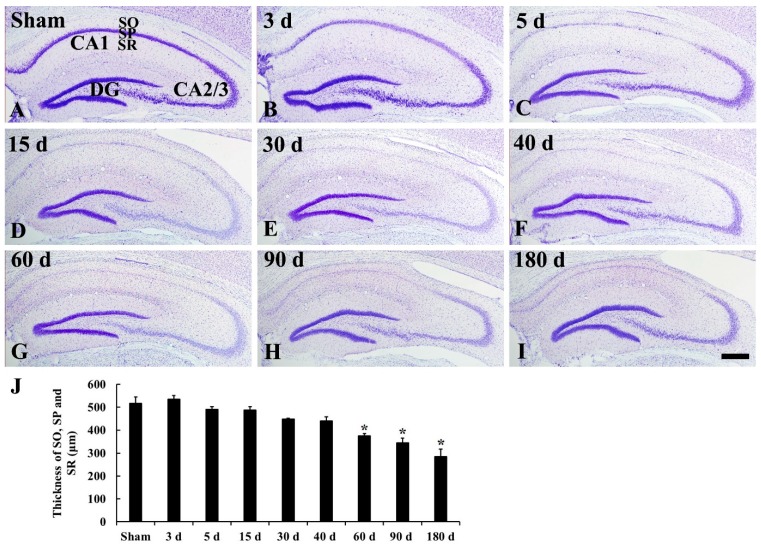
Cresyl violet (CV) staining in the hippocampus of the sham operated (**A**) and ischemia operated (**B**–**I**) groups after tgCI. CV stainability is decreased in the stratum pyramidale (SP) from 5 days after transient global cerebral ischemia (tgCI). In addition, the thickness (**J**) of damaged the hippocampal CA1 area (CA1) is significantly reduced from 60 days after tgCI. DG, dentate gyrus; SO, stratum oriens; SR, stratum radiatum. Scale bar = 50 μm. J: Thickness of SO, SP, and SR (*n* = 7; * *p* < 0.05 vs. sham operated group). The bars indicate the means ± SEM.

**Figure 2:**
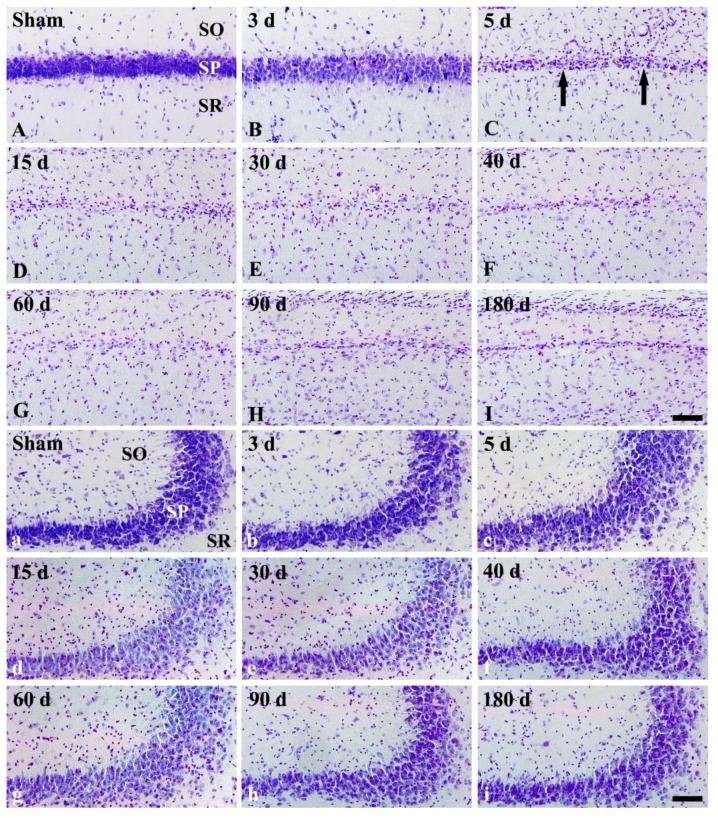
High magnification of CV staining of CA1 (**A**–**I**) and CA2/3 (**a**–**i**) of the sham operated (**A**,**a**) and ischemia operated (**B**–**I**, **b**–**i**) groups after tgCI. In the CA1, CV stained cells were hardly shown in the stratum pyramidale (SP, arrows) from 5 days after tgCI. In the CA2/3, CV^+^ cells in the SP are slightly pale from 15 days after tgCI compared to those in the sham operated group. SO, stratum oriens; SR, stratum radiatum. Scale bar = 50 μm.

In the sham operated group, pyramidal neurons, which are in the stratum pyramidale of the hippocampus proper (CA1–3), were well stained with cresyl violet (CV) (Figure 1A and Figure 2A,a). Three days after tgCI, CV staining was slightly brightened in pyramidal neurons in the CA1 due to reduced Nissl substance (Figure 1B and Figure 2B). Pyramidal neurons in the stratum pyramidale of the CA1, which are named CA1 pyramidal neurons, were apparently damaged (not stained with CV) from 5’ to 180 days after tgCI (Figure 1C–I and Figure 2C–I). In addition, the thickness of the CA1 was gradually reduced and significantly decreased compared to that in the sham operated group at 60, 90, and 180 days after tgCI (Figure 1G–J). 

On the other hand, CV positive (CV^+^) pyramidal neurons in the CA2/3 were not significantly changed after tgCI, although they were slightly brightened from 15 days after tgCI (Figure 2b–i).

### 2.2. FJB Histofluorescence Staining

**Figure 3:**
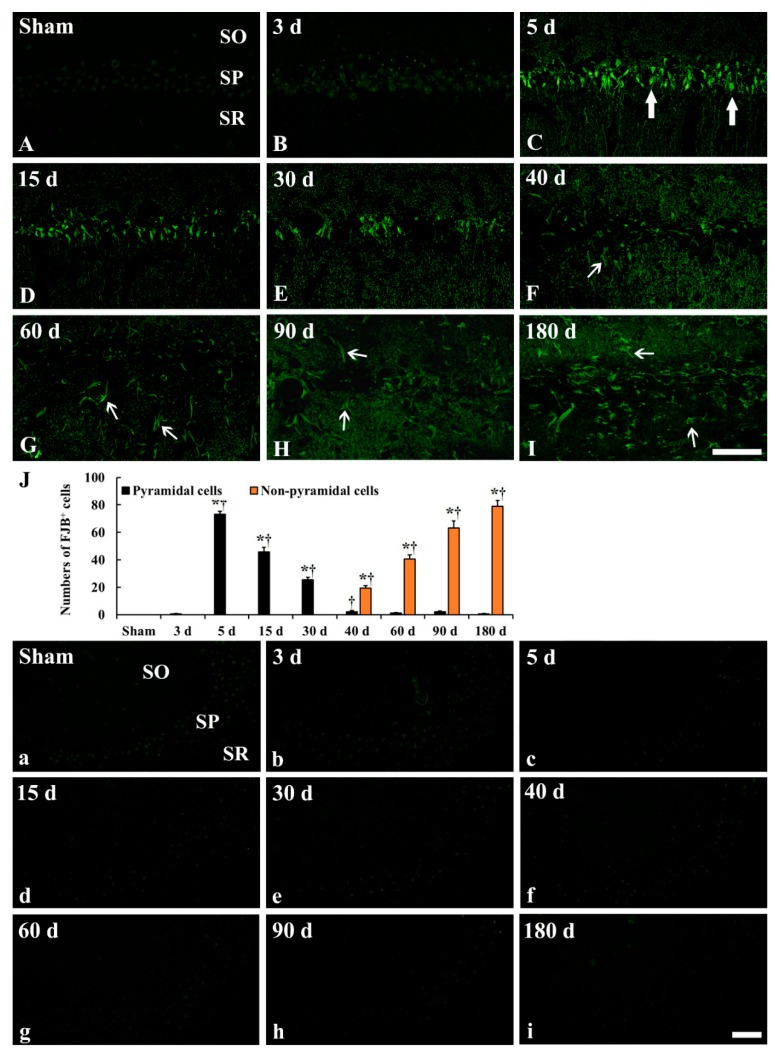
Fluoro-Jade B (FJB) fluorescence staining in the CA1 (**A**–**I**) and CA2/3 (**a**–**i**) of the sham operated (**A**,**a**) and ischemia operated (**B**–**I**, **b**–**i**) groups after tgCI. In the CA1, FJB^+^ pyramidal cells (large arrows) are shown in the stratum pyramidale (SP) from 5 to 30 days after tgCI. In addition, many FJB^+^ cells (small arrows) are detected in the stratum oriens (SO) and radiatum (SR) from 40 to 180 days after tgCI. In the CA2/3, FJB^+^ cells are not shown at any time after tgCI. Scale bar = 50 μm. (**J**) Numbers of FJB^+^ pyramidal (black) and non-pyramidal (yellow) cells in the CA1 (*n* = 7; * *p* < 0.05 vs. sham operated group, ^†^ *p* < 0.05 vs. pre-time point group). The bars indicate the means ± SEM.

In the CA1, FJB positive (FJB^+^) cells were not detected in any layers of the sham operated group (Figure 3A). In the ischemia operated group, FJB^+^ cells were not found until 3 days after tgCI (Figure 3B). However, a great number of pyramidal cells in the CA1 were stained with FJB (called FJB^+^ CA1 pyramidal cells) at 5 days after tgCI (Figure 3C), and such finding was observed until 30 days after tgCI (Figure 3D,E), showing that the number of FJB^+^ CA1 pyramidal cells was gradually decreased (Figure 3J). Thereafter, FJB^+^ CA1 pyramidal cells were hardly detected (Figure 3F–I). Instead, FJB^+^ non-pyramidal cells, which seemed to be astrocytes, were first detected in the strata oriens and radiatum of the CA1 at 40 days after tgCI (Figure 3F), thereafter, many FJB^+^ CA1 non-pyramidal cells were detected in all layers until 180 days after tgCI (Figure 3G–I). The number of FJB^+^ CA1 non-pyramidal cells was highest at 180 days after tgCI (Figure 3I,J). 

In the CA2/3, FJB^+^ cells were not detected in any layers of the sham operated group the CA2/3 region (Figure 3a). Similarly, in the ischemia operated groups, FJB^+^ cells were not observed in any layers of the CA2/3 at any time after tgCI (Figure 3b–i).

### 2.3. GFAP Immunoreactive Astrocytes

**Figure 4:**
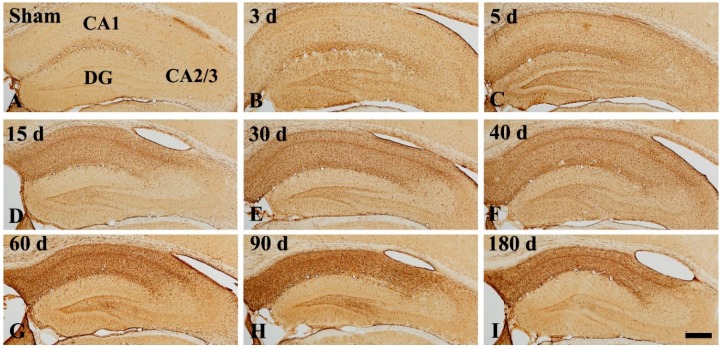
Low magnification of GFAP immunohistochemistry in the hippocampus of the sham operated (**A**) and ischemia operated (**B**–**I**) groups after tgCI. In the sham operated group, glial fibrillary acidic protein (GFAP) immunoreactivity is shown lower part of the CA1. From 3 days after tgCI, GFAP immunoreactivity in the CA1 is increased and very strong in all layers from 15 days post-tgCI. The immunoreactivity in the CA1 is highest at 90 days after tgCI. In the CA2/3, GFAP immunoreactivity is slightly increased at 3 and 5 days after tgCI. Scale bar = 400 μm.

**Figure 5:**
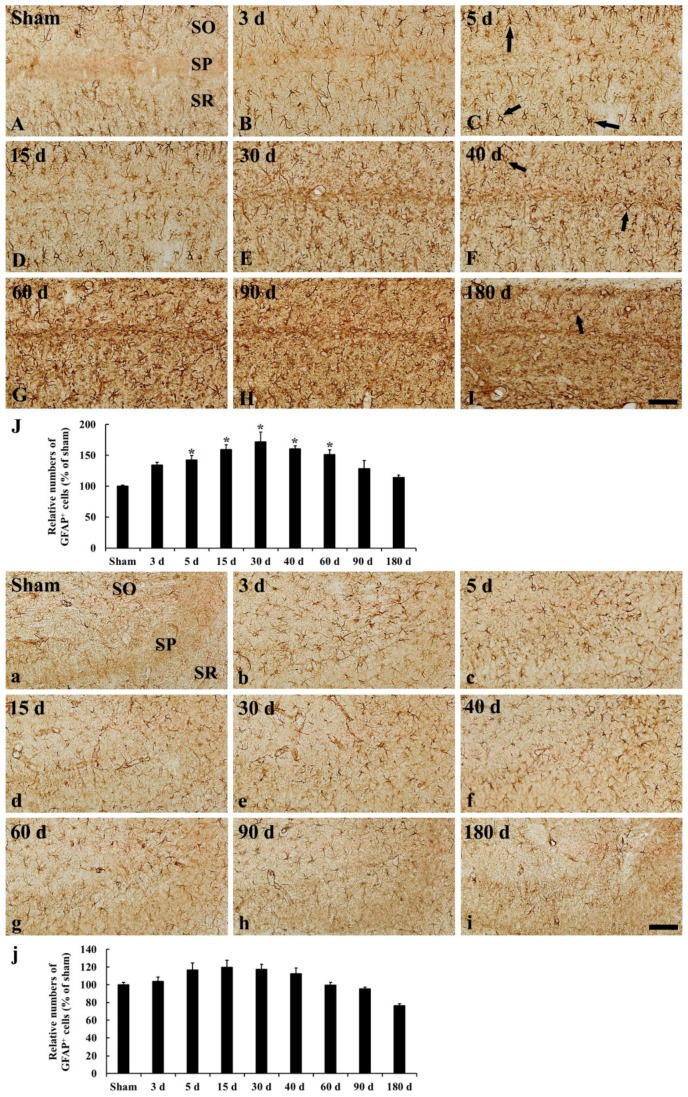
High magnification of GFAP immunohistochemistry in the CA1 (**A**–**I**) and CA2/3 (**a**–**i**) of the sham operated (**A**,**a**) and ischemia operated (**B**–**I**, **b**–**i**) groups after tgCI. In the sham operated group, GFAP immunoreactive astrocytes show typical resting form. In the ischemia operated group, GFAP immunoreactive astrocytes in the CA1 become larger in cell body and thicker in their processes until 40 days after tgCI. From 60 days after tgCI, GFAP immunoreactive astrocytes are broken in morphology, and the broken morphology is severest at 180 days after tgCI. In the CA2/3, GFAP immunoreactive astrocytes are not significantly altered in morphology after tgCI. Scale bar = 50 μm. (**J**,**j**) Relative numbers of GFAP immunoreactive astrocytes in the CA1 (**J**) and CA2/3 (**j**) (*n* = 7; * *p* < 0.05 vs. sham operated group). The bars indicate the means ± SEM.

In the CA1, GFAP immunoreactive (GFAP^+^) astrocytes in the sham operated group showed a stellate-shape, as a resting form, with small cell body and long and thin processes (Figure 4A and Figure 5A). In the ischemia operated group, GFAP^+^ astrocytes started to show morphological change. A few of them became bigger at 3 days after tgCI, showing that their processes became thicker (Figure 4B and Figure 5B). Between 5 and 15 days after tgCI, most of GFAP^+^ astrocytes showed hypertrophied cell bodies, and their processes became unevenly thicker and shorten (Figure 4C,D and Figure 5C,D). At these times, the number of GFAP^+^ astrocytes was significantly increased (1.43 and 1.59-fold, respectively, of the sham operated group) (Figure 5J). Thirty days after tgCI, cell bodies of GFAP immunoreactive astrocytes in the CA1 were still hypertrophied, and their processes were short with ragged edges (Figure 4E and Figure 5E). Currently, the number of GFAP immunoreactive astrocytes was highest (1.72 fold) (Figure 5J). From 40 days after tgCI, numbers of activated GFAP immunoreactive astrocytes were gradually decreased until 180 days after tgCI (Figure 5J). At 40 days after tgCI, the morphology of most of GFAP^+^ astrocytes was similar to that at 30 days tgCI, but some astrocytes showed broken line-like GFAP immunoreactivity (Figure 4F and Figure 5F). At 60 and 90 days after tgCI, all layers of the CA1 region were packed with hypertrophic GFAP^+^ astrocytes with thick and short processes, showing that a few of GFAP^+^ astrocytes showed small cell body with less-ramified processes (Figure 4G,H, Figure 5G,H). At 180 days after tgCI, many of GFAP^+^ astrocytes became smaller in the cell body, showing that broken processes were scattered around the cell bodies (Figure 4I and Figure 5I).

In the CA2/3, GFAP^+^ astrocytes of the sham operated group were similar to those in the CA1 (Figure 4A and Figure 5a). In the ischemia operated group, the morphology of GFAP^+^ astrocytes was slightly changed after tgCI. The cytoplasm of GFAP^+^ astrocytes became a bit larger, and their processes became a little thicker compared to those in the sham operated group at 3 and 5 days after tgCI (Figure 4B,C and Figure 5b,c). Thereafter, the morphology and distribution pattern of GFAP^+^ astrocytes in the CA2/3 were similar to that in the sham operated group (Figure 4D–I and Figure 5d–i), showing that the number of GFAP^+^ astrocytes was decreased (0.76 fold of the sham operated group) at 180 days after tgCI (Figure 5j). 

### 2.4. FJB^+^/GFAP^+^ Cells

**Figure 6:**
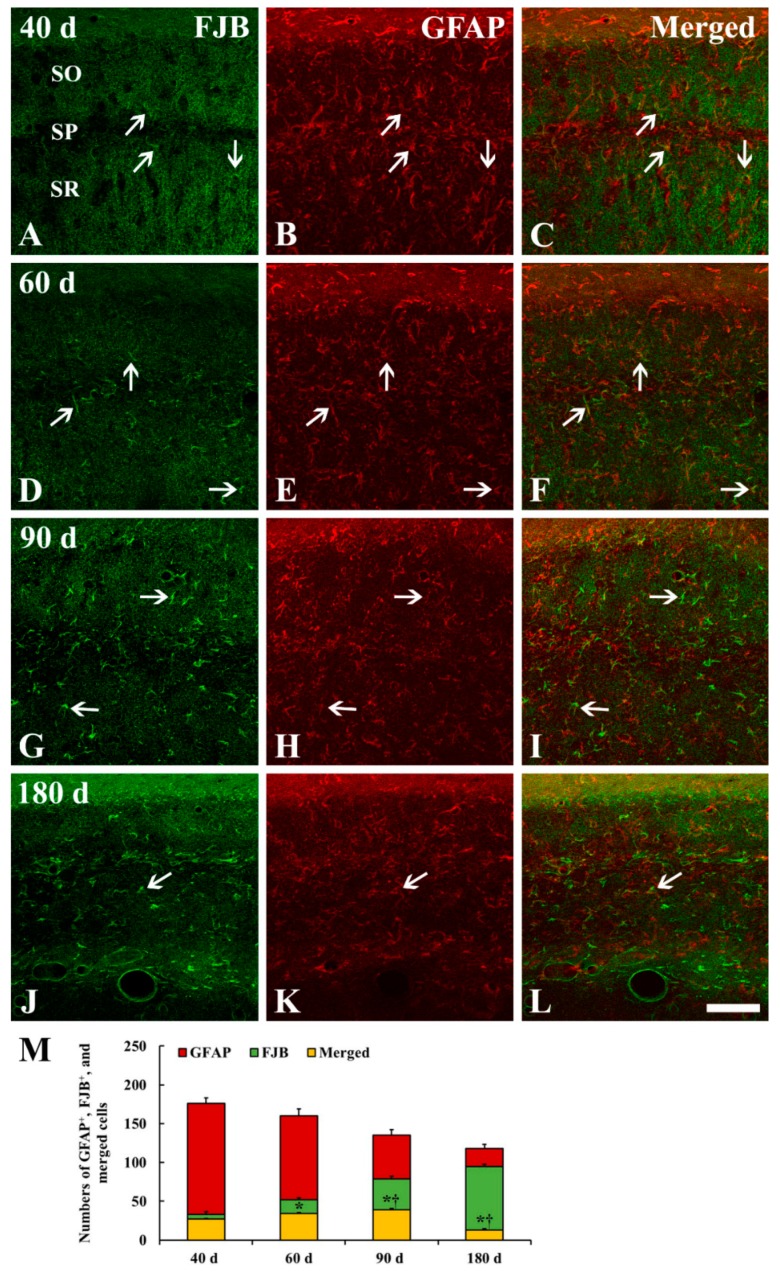
Double immunofluorescence staining for FJB (**A**,**D**,**G**,**J**), GFAP (**B**,**E**,**H**,**K**) and merged images (**C**,**F**,**I**,**L**) in the CA1 at 40 (**A**–**C**), 60 (**D**–**F**), 90 (**G**–**I**), and 180 (**J**–**L**) days after tgCI. The number of FJB^+^/GFAP^+^ astrocytes (degenerating astrocytes, white arrows) is significantly increased until 90 days after tgCI. Thereafter, the number is gradually decreased. On the contrary, the number of FJB^+^ cells (dead astrocytes) is gradually and significantly increased with time. Scale bar = 50 μm. (**M**) Numbers of GFAP^+^, FJB^+^, and FJB^+^/GFAP^+^ cells (*n* = 7; * *p* < 0.05 vs. sham operated group, ^†^ *p* < 0.05 vs. pre-time point group). The bars indicate the means ± SEM.

To specify the cell type of FJB^+^ cells which were distributed in the strata oriens and radiatum of the CA1 from 40 to 180 days after tgCI, we conducted double fluorescence staining for FJB and GFAP (Figure 6). Like the results of GFAP immunohistochemistry and FJB fluorescence staining, the number of GFAP^+^ astrocytes was gradually decreased, and the number of FJB^+^ cells was gradually increased in the CA1 after tgCI (Figure 6M). Simultaneously, the number of FJB and GFAP merged (FJB^+^/GFAP^+^) cells, which mean astrocytes and indicate degenerating astrocytes, was significantly increased by 90 days after tgCI. Thereafter, the number was significantly decreased at 180 days after tgCI (Figure 6M). The proportion of FJB^+^/GFAP^+^ astrocytes was 81.82, 65.38, 49.37, and 13.68% at 40, 60, 90, and 180 days after tgCI, respectively (Figure 6C,F,I,L,M). On the contrary, the number of FJB^+^ cells, which might indicate dead astrocytes, was significantly increased with time, showing that the proportion of FJB^+^ cells was 287.88% at 180 days after tgCI (Figure 6M).

## 3. Discussion

Until now, primary damages to neurons or astrocytes that occur immediately after tgCI has been well known. However, secondary pathologic events that occur over a long period following tgCI are not well studied. In the present study, we examined the long-term structural change of the hippocampus and morphological change of neurons and astrocytes in the gerbil hippocampal CA1-3 after 5 min of tgCI. 

### 3.1. Ischemic Damage of CA1 Pyramidal Neurons and Shrinkage of CA1 Area

In this study, pyramidal neuronal loss was restricted to the hippocampal CA1 area, and this degenerating process in CA1 pyramidal neurons (degeneration of CA1 pyramidal neurons) began from 5 days after tgCI and continued until 40 days after tgCI, and non-pyramidal cells in the strata oriens and radiatum showed remarkable shrinkage from 60 days after tgCI. It is well known that obvious neuronal death occurred in the pyramidal cell layer in the CA1 area of the gerbil at 4 to 5 days after tgCI [1,2]. In addition, Lee et al. (2010) recently reported that the death of CA1 pyramidal cells lasted until about 45 days after tgCI in the gerbil hippocampus following 5 min of tgCI [20]. Based on the previous and our present studies, it is likely that pyramidal cell death in the CA1 area after tgCI is a progressive event over a long period following tgCI.

We found in this study that the thickness of the CA1 region was gradually and significantly reduced at 60, 90, and 180 days after tgCI compared to that in the sham operated group. It has been reported that the shrinkage of CA1 area is shown at 6 months after cerebral ischemia in rats due to the loss of pyramidal neurons in the stratum pyramidale, damaged apical dendrites of pyramidal neurons, and retracted dendrites of GABAergic interneurons in the stratum radiatum, and depleted fiber bundles in the stratum lacunosum moleculare in the CA1 area in the rat hippocampus [21]. These findings indicate that tgCI-induced neurodegeneration develops significant atrophy in the brain.

### 3.2. Numerical and Morphological Changes of GFAP^+^ Astrocytes in Ischemic Hippocampus

In this study, there was a significant increase in the number of GFAP^+^ astrocytes in the CA1 area, but not in the CA2/3 area, from 5 to 30 days after tgCI. This finding is in partial agreement with previous studies that showed that GFAP mRNA was dramatically increased in the hippocampus in the first few days after 10 min of tgCI in gerbils [22] and that morphologically activated astrocytes began to increased 7 days after 10 min of global cerebral ischemia, showing that weakly reactive astrocytes were observed in the CA3 region in rats [10,23]. Furthermore, the number of hypertrophic GFAP^+^ cells was significantly increased from 7 days after ischemia, and the number persisted until 21 days after ischemia in rats [24]. On the other hand, in this study, we found that the number of GFAP^+^ astrocytes in the ischemic CA1 region gradually declined from 40 days after tgCI. It has been reported that the number of GFAP^+^ astrocytes was significantly reduced in the CA1 region at 28 to 56 days after 10 min of tgCI in rats [4] and at 14 to 30 days after 7 min of tgCI in gerbils [25]. These results indicate that astrocytes might respond to ischemia in a different manner according to duration after transient ischemia, which is related to different neuronal vulnerability after tgCI. 

In addition, our results showed that the morphology of GFAP immunoreactive astrocytes in the ischemic CA1 region was continuously changed until 180 days after tgCI. In particular, GFAP^+^ astrocytes were severely damaged (shrunken or destroyed) and showed strong GFAP immunoreactivity between 60 and 180 days after tgCI: At these times, the CA1 area was significantly reduced in thickness. It has been reported that a subpopulation of astrocytes in peri-infarct area is activated and continually synthesized GFAP until the end of glial scar formation [26,27]. Furthermore, glial scar gradually narrows as it matures over many months and it is characterized by persistence of increased GFAP immunoreactivity and tightly interwined astrocytic processes [28]. Based on these findings, our present finding indicates that, at late stages after ischemia, astrogliosis forms a glial scar around the region in which ischemic neuronal death occurs.

### 3.3. Degeneration/Death of GFAP^+^ Astrocytes in Ischemic Hippocampus

During glial scar formation, the death of astrocytes has been demonstrated so far in animal models of focal cerebral ischemia. It has been reported that astrocytes undergo ischemia-induced programmed cell death in the penumbra zone of ischemic cortex after transient focal ischemia in mice or rats by detecting TUNEL positive DNA fragmentated astrocytes [11,12,29] or caspase-12 positive nuclear-condensed astrocytes [11,30]. To the best of knowledge, there are few studies on degeneration or death of astrocytes after tgCI by using double immunofluorescence staining for GFAP and FJB. In the present study, we found that FJB/GFAP^+^ astrocytes began to appear in the CA1 region at 40 days after tgCI, and the number of FJB/GFAP^+^ astrocytes was significantly increased until 90 days after tgCI and decreased at 180 days after tgCI. At this time, only FJB^+^ cells were dramatically increased in the ischemic CA1 region. This is the first study that showed that the death of astrocytes in the ischemic CA1 region occurred from at least 60 days after 5 min of tgCI. For reference, Damjanac et al. (2007) have reported that, in APP_SL_/PS1KI transgenic mice of Alzheimer’s disease, FJB^+^ cells are colocalized with hypertrophic GFAP^+^ astrocytes in the CA1 region at 6 to 10 months following a marked reduction of pyramidal neurons, suggesting that FJB could label activated astrocytes during a chronic neuronal degenerative process [31]. Taken together, our present finding suggests that subsequent degeneration or death of astrocytes in the CA1 region begins at 40 days after tgCI and continues until 180 days (about 6 months) after tgCI. In addition, we insist that FJB can be used to label degenerating or dead astrocytes during a chronic degenerative period following tgCI. 

### 3.4. Summary

In summary, 5 min of tgCI induced a progressive death of pyramidal neurons accompanied by reactive astrogliosis in the hippocampal CA1 region until 30 days after tgCI. Thereafter, CA1 pyramidal cell death disappeared, and astrocytes might participate in forming a glial scar via degeneration of death of astrocytes. Finally, the ischemic CA1 region was shrunken in thickness. These suggest that astrocyte reaction is closely related to neuronal damage or death after ischemic insults and astrocytes are degenerated or dead to form scar in damaged regions. 

## 4. Materials and Methods

### 4.1. Experimental Animals

Male Mongolian gerbils (total *I* = 96) were obtained at 6 months of age (body weight, 70–75 g) from the Experimental Animal Center (Kangwon University, Chuncheon, Gangwon, Korea) and kept at a constant temperature (23 °C) and humidity (50%) with a 12-h light/dark cycle. The process of the care and handling of the gerbils conformed to the guidelines following current international laws and policies (NIH Guide for the Care and Use of Laboratory Animals, The National Academies Press, 8th Ed., 2011). The protocol of this experiment was approved by the Institutional Animal Care and Use Committee (IACUC) at Kangwon National University on 24 January 2018 (approval no. KW-180124-1).

### 4.2. tgCI Induction

As described in our published paper [32], the induction of tgCI in gerbils was performed. In brief, the gerbils were anesthetized with a mixture of isoflurane (2.5%) in oxygen (33%) and nitrous oxide (67%), and bilateral common carotid arteries were occluded for 5 min by using aneurysm clips. The complete interruption of blood flow was confirmed by observing the central artery in the retina by using an ophthalmoscope (Heine Optotechnik, Herrsching am Ammersee, Germany). Body temperature was kept (37 ± 0.2 °C) by using a thermometric blanket during tgCI and until they had recovered from anesthesia. Sham operated gerbils were subjected to the same procedure without bilateral common carotid artery occlusion.

### 4.3. Tissue Preparation for Histology

For cresyl violet (CV) staining, Fluoro-Jade B (FJB) histofluorescence, immunohistochemical and double immunofluorescence staining, sections containing the hippocampus were prepared from the sham (*n* = 5 at each time) and tgCI operated gerbils (*n* = 7 at each time) at 3, 5, 15, 30, 40, 60, 90, and 180 days after tgCI. According to our published method [32], in brief, the gerbils were anaesthetized with 60 mg/kg sodium pentobarbital (JW Pharm. Co., Ltd., Seoul, Korea) and fixed transcardially with 4% paraformaldehyde. Their brains were removed and postfixed with the same fixative for 7 h and cryoprotected by infiltration with 30% sucrose for 10 h. The tissues were serially sectioned into 30-μm frontal sections in a cryostat (Leica, Wetzlar, Germany).

### 4.4. CV Staining

To investigate cellular distribution and morphology, CV staining was performed according to our published protocol [32]. In brief, 1.0% (*w*/*v*) CV acetate (Sigma, Darmstadt, Germany) solution was prepared, and 0.28% glacial acetic acid (Sigma) was added to this solution. The sections were stained with the solution.

### 4.5. FJB Histofluorescence Staining

To investigate the degeneration/death of cells, FJB (a fluorescent marker for the localization of cellular degeneration) histofluorescence staining was conducted according to the method published by Candelario-Jalil et al. [33]. In brief, the sections were immersed in 1% sodium hydroxide in 80% alcohol and followed in 70% alcohol. They were then transferred to 0.06% potassium permanganate solution and incubated in 0.0004% FJ B (Histochem, Jefferson, AR, USA) solution. Finally, they were placed on a slide warmer (about 50 °C) to be reacted. The reacted sections were examined using an epifluorescent microscope (Carl Zeiss, Göttingen, Germany), which was equipped with blue excitation light (450–490 nm).

### 4.6. Immunohistochemistry

To examine the distribution and morphology of astrocytes, immunohistochemistry was done according to our published method [34]. In short, the sections were sequentially treated with 0.3% hydrogen peroxide (H_2_O_2_) for 30 min and 10% normal donkey serum for 30 min. The treated sections were incubated with mouse anti-GFAP (1:800, Abcam, Cambridge, England) overnight at 4 °C. The incubated sections were exposed to biotinylated horse anti-mouse immunoglobulin G (IgG) (1:200, Vector, Burlingame, CA, USA) and streptavidin peroxidase complex (1:200, Vector). Finally, the reacted sections were visualized with 3,3’-diaminobenzidine tetrahydrochloride. 

A negative control test was done to establish the specificity of GFAP immunostaining with pre-immune serum instead of mouse anti-GFAP. The negative control test showed no immunostaining (data not shown).

### 4.7. Double Fluorescence Staining

To examine the kind of cell that showed FJB fluorescence staining, double fluorescence staining was performed according to published protocol [31,35]. In brief, the sections were reacted with mouse anti-GFAP (1:200, Abcam) for astrocytes like above-mentioned method, and the GFAP-reacted sections were reacted in goat anti-mouse IgG, Alexa Fluor 546 (1:500, Invitrogen, Waltham, MA, USA). Subsequently, the GFAP-reacted sections were subjected to FJC staining as described above, except the time in potassium permanganate solution was reduced to 5 min not to alter the immunofluorescent GFAP labeling. The double immunoreaction was observed using a confocal MS (LSM510 META NLO, Carl Zeiss).

### 4.8. Data Analysis

First, we analyzed the thickness of the hippocampus because damaged brain areas must be significantly atrophied, according to the previously published method [36]. In brief, we selected the CV stained coronal sections at the level Bregma −2 mm of the gerbil brain atlas [37] and measured the thickness of hippocampus (strata oriens, pyramidale, and radiatum in the CA1 region) at the point set at 1.5 mm from the center. 

Secondly, numbers of GFAP^+^ and FJB^+^ cells were analyzed according to our published method [38]. In brief, we selected five sections from each animal with 120-μm interval at the level of −1.65 to −2.7 mm of the gerbil brain atlas [37]. We captured images of GFAP^+^ and FJB^+^ cells in the hippocampus by using an AxioM1 light microscope (Carl Zeiss): The positive cells were obtained in a 250 × 250 µm square. Cell count was done by averaging the total number of GFAP^+^ and FJB^+^ cells by using an Adobe Photoshop (version 8.0) and Image J (1.46 software) (National Institutes of Health, Bethesda, MD, USA).

### 4.9. Statistical Analysis

The data represent means ± SEM. Differences of the means among the groups were statistically analyzed by analysis of variance (ANOVA) with Duncan’s post hoc test by using SPSS 17.0 software (IBM, New York, NY, USA). Statistical significance was considered at *p* < 0.05.
